# Does walkable neighbourhood design influence the association between objective crime and walking?

**DOI:** 10.1186/s12966-014-0100-5

**Published:** 2014-07-26

**Authors:** Sarah Foster, Matthew Knuiman, Karen Villanueva, Lisa Wood, Hayley Christian, Billie Giles-Corti

**Affiliations:** 1 Centre for the Built Environment and Health, School of Population Health, The University of Western Australia, 35 Stirling Highway, Crawley 6009, WA, Australia; 2 McCaughey VicHealth Centre for Community Wellbeing, Melbourne School of Population and Global Health, University of Melbourne, Melbourne 3010, Victoria, Australia; 3 Telethon Institute for Child Health Research, The University of Western Australia, 35 Stirling Highway, Crawley 6009, WA, Australia

**Keywords:** Crime, Walking, Walkability, Destinations, Built environment, Adults

## Abstract

**Background:**

Few studies have investigated associations between objectively measured crime and walking, and findings are mixed. One explanation for null or counterintuitive findings emerges from criminology studies, which indicate that the permeable street layouts and non-residential land uses that underpin walkable neighbourhoods are also associated with more crime. This study examined associations between objective crime and walking, controlling for the characteristics of walkable neighbourhoods.

**Methods:**

A population representative sample of adults (25–65 years) (n = 3,487) completed the Western Australian Health and Wellbeing Survey (2006–2008) demographic and walking frequency items. Objective environmental measures were generated for each participant’s 400 m and 1600 m neighbourhood areas, including burglary, personal crime (i.e., crimes committed against people) in public space, residential density, street connectivity and local destinations. Log-linear negative binomial regression models were used to examine associations between crime and walking frequency/week, with progressive adjustment for residential density, street connectivity and local destinations.

**Results:**

Burglary and personal crime occurring within a participant’s 400 m and 1600 m neighbourhoods were positively and significantly associated with walking frequency. For example, for every additional 10 crimes against the person/year within 400 m of a participant’s home, walking frequency increased by 8% (relative change = 1.077, *p* = 0.017). Associations remained constant after controlling for residential density and street connectivity, but attenuated after adjusting for local destinations (e.g., for personal crime in 400 m: relative change = 1.054, *p* = 0.104). This pattern of attenuation was evident across both crime categories and both neighbourhood sizes.

**Conclusions:**

The observed positive associations between objective crime and walking appear to be a function of living in a more walkable environment, as the presence of destinations has the capacity to both promote walking and attract crime. This study provides a plausible explanation for some mixed findings emerging from studies examining crime as a barrier to walking. In some settings, the hypothesised deterrent effect of crime on walking may be insufficient to outweigh the positive impacts of living in a more walkable environment.

## Background

In recent years, studies investigating the neighbourhood influences on physical activity have proliferated [1]–[3]. For some neighbourhood attributes, such as higher residential densities, street connectivity and access to destinations, the accumulated evidence is sufficient to warrant public health and planning bodies to advocate for changes to the built environment as a means of increasing walking [4]–[6]. However, the evidence for other neighbourhood attributes, such as crime, is mixed, rendering it difficult to draw a definitive conclusion about its impact on physical activity [7].

The general assumption in physical activity studies is that neighbourhoods with more crime will cause people to feel unsafe or fearful, and that this will negatively impact physical activity levels, particularly activities that take place in the streets and public spaces [7]–[9]. Given this prevailing assumption, it is relatively common for studies examining the impact of the environment on physical activity to include some measure of crime-related safety, such as general perceptions of safety [10], judgements about crime [11], fear of crime [12],[13], visual indicators of crime (e.g., graffiti, vandalism) [14], or ‘objective’ crime rates [15]. However, these measures are not necessarily interchangeable as representations of the same construct. For example, studies have documented limited agreement between ‘perceived’ and ‘objective’ measures of crime [9],[16]–[18], suggesting that they may capture different elements of the neighbourhood environment [17],[18].

Despite the number of studies examining crime-related safety and physical activity, relatively few have investigated the association between objective crime and physical activity, and again, the findings are mixed. Several studies support the hypothesis that higher objective crime rates constrain walking [15],[17],[19] and physical activity [20]–[24]. Moreover, there is some evidence indicating that violent crime (e.g., murder, robbery) may have a greater impact on walking behaviour than property (e.g., burglary, theft, vandalism) or quality of life crimes (e.g., prostitution, drug offences) [15]. Conversely, others have reported no association between objective crime rates and walking [18],[25],[26] or physical activity [27],[28], or counterintuitive positive associations [29]. For instance, higher ‘person crime’ (i.e., crimes against people, excluding those of a sexual nature) was positively associated with walking frequency in deprived Glasgow neighbourhoods [29]. This association attenuated with further adjustment, however it serves to highlight the mixed nature of the evidence linking crime and walking. Indeed, as identified elsewhere [30], low income populations may have little choice but to walk, or may be desensitised to local crime levels [29].

An alternative explanation for these null or counterintuitive associations is that the characteristics of more walkable neighbourhoods also tend to create more opportunities for crime [31],[32]. For instance, the non-residential land uses that provide destinations to walk to (e.g., shopping centres, recreational facilities and transport nodes) have been associated with higher levels of property crime [33]–[36], and the presence of drinking venues and alcohol sales linked with more violent crime [37]–[39]. Similarly, street connectivity is integral to a walkable neighbourhood as it provides both direct and varied walking routes for residents [40]. However, better connected streets (i.e., gridded street layouts) also ensure the neighbourhood is more easily navigated by would-be offenders, with more potential ‘escape routes’ [34]. Indeed, the consensus from much of the criminology literature is that higher street connectivity increases vulnerability to crime [31],[41],[42]. Just as the combined presence of several ‘walkable’ attributes facilitates walking, their cumulative presence may also be most pertinent to crime. For example, permeable streets may not impact crime unless destinations are present that draw potential offenders to an area [34].

Could the intrinsic qualities of walkable neighbourhoods account, at least in part, for some of the null or unexpected positive findings between crime and physical activity? The aim of this study was to examine the association between objective measures of neighbourhood crime (i.e., crimes reported to police) and walking frequency for a population representative sample of adults in Perth, Western Australia. Furthermore, we were interested in examining whether the presence of walkable neighbourhood attributes, such as residential density, street connectivity and local destinations, affected the associations between crime and walking.

## Methods

### Study participants and setting

This study forms part of the Life Course Built Environment and Health project, a cross-sectional data linkage study exploring associations between built environment features and health across different life stages in Perth, Western Australia [43]. Participants were a stratified random sample drawn from the Perth metropolitan area who completed the Western Australian Health and Wellbeing Surveillance System (HWSS) survey from 2003–2009 (n = 21,347) administered by the Department of Health of Western Australia (DoHWA). For survey participants who permitted linkage to other datasets, objectively-measured environment variables were calculated. Overall 74.7% consented to data linkage and had a geocoded home address (n = 15,954). Ethics approval was obtained from DoHWA and The University of Western Australia (ref 2010/1). As crime location data was only available for the 2007 calendar year, this study focuses on adults, aged 25–65 years, who completed the HWSS survey between 2006 and 2008 (n = 3,487).

### Definition of neighbourhood

Detailed information on the methods used to develop spatial environment variables is published elsewhere [43]. Briefly, Geographic Information Systems (GIS) software (ArcGIS v10) was used to measure the attributes of each participant’s ‘neighbourhood’ based on the road network service area around their home. For the purposes of this study, we focused on the 400 m and 1600 m ‘neighbourhoods’. A 1600 m service area is typically used in studies with adults, as this represents how far they could walk from home at moderate to vigorous intensity within 15 minutes, which is half of the recommended level of daily physical activity for adults [44]. However, we were also interested in the association between crime and walking in the more proximate 400 m environment, as offences closer to home may have a bigger impact on residents’ behaviour.

### Measures of neighbourhood crime and built environment attributes

The Western Australia Police supplied the spatial locations of reported crimes for the 2007 calendar year, divided into: (1) actual and attempted burglary; and (2) personal crime in public space (i.e., crimes committed against people such as threats, disorderly behaviour, assault and robbery). For each crime category, the count of offences within 400 m and 1600 m of participants’ home addresses were calculated. Local retail and service destinations (e.g., deli, shops, pharmacy, fast food restaurant, bank etc.) were obtained from a database of commercial destinations (i.e., SENSIS Pty Ltd) and matched as closely as possible to year of survey completion [43]. The total number of local destinations, and a sub-set of destinations that potentially serve alcohol (i.e., hotels, pubs, clubs and restaurants), were calculated for the 400 m and 1600 m service areas around each participant’s home. The sub-set of alcohol-related destinations was chosen because of the established relationship between alcohol sales and violent crime [38]. Residential density was calculated as the ratio of residential dwellings to residential area in hectares, and street connectivity as the ratio of three-way intersections to the service area [43].

### Socio-demographic adjustment variables

These included sex, age in years, marital status (partner, no partner) and indicators of individual socio-economic status such as educational attainment (secondary or less, Technical and Further Education (TAFE) or trade qualification, university degree or equivalent), and area-level socio-economic status (i.e., using the Index of Relative Socio-Economic Disadvantage (IRSD) for the corresponding census collection districts) [45].

### Walking outcome

The study outcome was self-reported walking frequency per week. As part of the HWSS, participants reported the number of times they walked in the past week (continuous).

### Statistical analyses

All analyses were conducted in SPSS v21. Negative binomial models (with log link) were used to compute the percentage change in walking frequency per one unit increase in the exposure variables. A log link allows the relative effect of variables on walking frequency to be estimated (i.e., percentage change in walking frequency). Negative binomial models are an appropriate choice because walking frequency is non-negative, positively skewed with an excess of zeroes (i.e., non-walkers), and has a variance that exceeds its mean.

Preliminary analyses examined the associations between the socio-demographic variables and walking frequency (Table 1), and between objective crime and built environment measures and walking frequency, with adjustment for socio-demographics (Table 2). Next, the typical characteristics of a more walkable neighbourhood (i.e., local destinations, residential density and street connectivity) were included in multivariable models in order to highlight any independent associations with walking frequency (Table 3).

**Table 1:** Sample characteristics and associations with walking frequency/week in adults 25–65 years (n = 3,487)

**Characteristics**	**%**	**Times walked/week mean (SD)**	P*
**Sex**			0.715
Male	38.2	3.84 (4.34)	
Female	61.8	3.89 (3.71)	
**Partner**			0.253
Yes	73.5	3.82 (3.76)	
No	26.4	4.00 (4.46)	
**Education**			**0.000**
Secondary or less	32.5	3.56 (3.65)	
Trade or certificate	40.8	3.75 (3.95)	
Tertiary	26.8	4.44 (4.26)	
**Mean age** (SD)	47.4 (10.5)		0.267
**Mean IRSD** (SD)	1030.1 (81.7)		**0.000**

*From negative binomial log-linear regression models. Bold denotes p < 0.05.

**Table 2:** Crime and built environment characteristics and their associations walking frequency/week in adults 25–65 years (n = 3,487)

**Spatial variable**	**Mean (SD)**	**Relative change (CI)**^**1**^	***p***
***Individual models***			
Burglary (400 m)	5.72 (6.47)	1.086 (1.030-1.145)	**0.002**
Burglary (1600 m)	93.54 (80.60)	1.007 (1.003-1.011)	**0.002**
Personal crime in public space (400 m)	1.46 (5.75)	1.077 (1.013-1.145)	**0.017**
Personal crime in public space (1600 m)	32.43 (68.46)	1.010 (1.004-1.016)	**0.001**
Residential density (400 m)^2^	11.86 (27.65)	0.999 (1.000-1.001)	0.375
Residential density (1600 m)^2^	12.73 (8.27)	1.004 (1.000-1.009)	0.066
Street connectivity (400 m)^3^	61.96 (30.27)	1.000 (1.000-1.001)	0.454
Street connectivity (1600 m)^3^	56.85 (18.90)	1.002 (1.000-1.003)	0.078
Local destinations (400 m)^4^	3.94 (10.58)	1.004 (1.001-1.007)	**0.015**
Local destinations (1600 m)^4^	80.06 (104.52)	1.001 (1.000-1.001)	**0.000**
Hotels, pubs, clubs & restaurants (400 m)^5^	0.23 (0.88)	1.057 (1.016-1.100)	**0.006**
Hotels, pubs, clubs & restaurants (1600 m)^5^	1.55 (8.56)	1.008 (1.004-1.012)	**0.000**

^1^From negative binomial log-linear models and represents change in walking frequency per unit increase in the spatial variable, except for burglary/personal crime variables where they represent change per increase of 10/year. All models adjust for age, sex, marital status, education and IRSD. ^2^Residential density calculated as the ratio of residential dwellings to residential area in hectares. ^3^Street connectivity calculated as the ratio of three-way intersections (or more) to the service area. ^4^Local destinations calculated as the count of all retail and service destinations in the service area. ^5^Subset of local destinations that are likely to serve alcohol. Bold denotes p < 0.05.

**Table 3:** Walkable neighbourhood characteristics and their associations walking frequency/week in adults 25–65 years (n = 3,487)

**Variable**	**Model 1**		**Model 2**	
	**Relative change (CI)**^**1**^	***p***	**Relative change (CI)**^**1**^	***p***
**400 m service area**				
Residential density	0.999 (0.997-1.001)	0.282	0.999 (0.997-1.001)	0.275
Street connectivity	1.000 (0.999-1.001)	0.860	1.000 (0.999-1.001)	0.831
Local destinations	1.004 (1.001-1.007)	**0.018**		
Hotels, pubs, clubs & restaurants			1.058 (1.016-1.102)	**0.006**
**1600 m service area**				
Residential density	1.000 (0.995-1.005)	0.954	1.000 (0.994-1.005)	0.871
Street connectivity	1.000 (0.998-1.002)	0.977	1.000 (0.998-1.003)	0.668
Local destinations	1.001 (1.000-1.001)	**0.001**		
Hotels, pubs, clubs & restaurants			1.008 (1.003-1.013)	**0.001**

^1^From negative binomial log-linear models and represents change in walking frequency per unit increase in the spatial variable. Model 1 includes residential density, street connectivity and local destinations. Model 2 includes residential density, street connectivity and a subset of local destinations that are likely to serve alcohol. All models adjust for age, sex, marital status, education and IRSD. Bold denotes p < 0.05.

Finally, a series of multivariable models were run to explore which walkable built environment characteristics would influence the association between crime and walking frequency (Table 4), as follows: Model 1 outlines the association between crime and walking frequency, controlling for socio-demographic variables only; Model 2 further adjusts for residential density and street connectivity; and Models 3a and 3b document the association between crime and walking frequency, adjusting for socio-demographic variables and local destinations (Model 3a) or destinations likely to serve alcohol (Model 3b).

**Table 4:** Associations between crime and walking frequency/week, with adjustment for different built environment characteristics, in adults 25–65 years (n = 3,487)

**Variable**	**Model 1**		**Model 2**		**Model 3a**		**Model 3b**	
	**Crime**		**Crime + residential density + street connectivity**		**Crime + local destinations**		**Crime + hotels, pubs, clubs & restaurants**^**1**^	
	**Relative change (CI)**^**1**^	***p***	**Relative change (CI)**^**1**^	***p***	**Relative change (CI)**^**1**^	***p***	**Relative change (CI)**^**1**^	***p***
***Actual and attempted burglary***								
**400 m service area**								
Burglary	1.086 (1.030-1.145)	**0.002**	1.087 (1.029-1.148)	**0.003**	1.069 (1.008-1.132)	**0.025**	1.063 (1.004-1.127)	**0.037**
Residential density			0.999 (0.997-1.001)	0.279				
Street connectivity			1.000 (0.999-1.001)	0.822				
Local destinations					1.002 (0.999-1.006)	0.179		
Hotels, pubs, clubs & restaurants^2^							1.039 (0.996-1.084)	0.076
**1600 m service area**								
Burglary	1.007 (1.003-1.011)	**0.002**	1.006 (1.001-1.011)	**0.011**	1.003 (0.997-1.008)	0.344	1.003 (0.998-1.008)	0.293
Residential density			1.000 (0.995-1.006)	0.861				
Street connectivity			1.001 (0.999-1.003)	0.468				
Local destinations					1.001 (1.000-1.001)	**0.015**		
Hotels, pubs, clubs & restaurants^2^							1.006 (1.001-1.012)	**0.016**
***Personal crime in public space***								
**400 m service area**								
Personal crime	1.077 (1.013-1.145)	**0.017**	1.078 (1.013-1.001)	**0.018**	1.054 (0.989-1.124)	0.104	1.049 (0.986-1.116)	0.131
Residential density			0.999 (0.997-1.001)	0.268				
Street connectivity			1.000 (0.999-1.001)	0.614				
Local destinations					1.003 (0.999-1.006)	0.114		
Hotels, pubs, clubs & restaurants^2^							1.045 (1.001-1.090)	**0.043**
**1600 m service area**								
Personal crime	1.010 (1.004-1.016)	**0.001**	1.009 (1.003-1.015)	**0.003**	1.006 (0.999-1.012)	0.098	1.004 (0.996-1.014)	0.326
Residential density			1.000 (0.995-1.005)	0.984				
Street connectivity			1.001 (0.999-1.003)	0.469				
Local destinations					1.000 (1.000-1.001)	**0.041**		
Hotels, pubs, clubs & restaurants^2^							1.005 (0.998-1.012)	0.139

^1^From negative binomial log-linear models and represents change in walking frequency per unit increase in the spatial variable, except for burglary/personal crime variables where they represent change per increase of 10/year. All models adjust for age, sex, partner, education and IRSD. ^2^Subset of destinations that are likely to serve alcohol. Bold denotes p < 0.05.

## Results

Higher education and area-level socio-economic status were positively associated with walking frequency (Table 1). Furthermore, all objective crime and destinations variables were, when considered individually, positively and significantly associated with walking frequency for both the 400 m and 1600 m neighbourhood (Table 2). For example, for every increase of ten personal crimes/year within 400 m, walking frequency increased by almost 8% (relative change 1.077, p = 0.017). This pattern, whereby crime was positively associated with walking frequency, was evident across both crime categories and both the 400 m and 1600 m neighbourhood service areas. The positive associations between destinations and walking frequency were also confirmed by a series of multivariable models that included other characteristics of a walkable neighbourhood (i.e., residential density and street connectivity) (Table 3).

Table 4 presents the associations between objective crime and walking frequency with adjustment for walkable built environment characteristics for both the 400 m and 1600 m service areas. Notably, there was minimal attenuation of the positive associations between either of the crime categories and walking frequency when residential density and street connectivity were included (Model 2). However, the observed associations between crime and walking weakened when the destinations variables were added to the models (Models 3a and 3b). For example, the relative change in walking frequency for each increase of ten personal crimes/year within 400 m attenuated from 1.077 (*p* = 0.017) in Model 1 to 1.054 (*p* = 0.104) in Model 3a. Indeed, when local destinations were substituted with the number of hotels, pubs, clubs and restaurants, there was even greater attenuation of the associations between both categories of crime and walking frequency (relative change = 1.049, *p* = 0.131) (Model 3b). This pattern of attenuation was largely consistent for both burglary and personal crime in public space, and for the 400 m and 1600 m service areas. Additional analyses confirmed moderate to strong correlations between objective crime and the destinations variables (i.e., most Pearson correlations were between 0.350 and 0.584, all *p* values = 0.01).

## Discussion

This study drew on the criminology literature to investigate the assumption that reported or ‘objective’ crime presents a barrier to residents’ walking. Somewhat counter-intuitively, we found a positive association between crime and walking, which is at odds with many of the studies examining reported crime and physical activity [15],[17],[19]–[21],[24]. However, our findings also provide a plausible explanation for other studies that document null or counterintuitive associations between crime and physical activity [18],[25]–[28],[46].

This study suggests that the destinations that underpin a more walkable neighbourhood are associated with both walking frequency and crime, but importantly, in these neighbourhoods crime does not appear to deter walkers. Specifically, we found that both burglary and personal crimes were positively associated with walking, but attenuated after adjusting for key characteristics of a walkable neighbourhood. While residential density and street connectivity had little attenuating influence, the presence of local destinations, and more specifically, destinations that potentially serve alcohol, appeared to explain the association between crime and walking. This pattern was largely consistent for both the 400 m and 1600 m ‘neighbourhood’ service areas, although there was less attenuation of the association between burglary and walking for the 400 m area.

It is well recognised that the presence of destinations required for daily living are vital to creating a healthy, walkable environment [47], but paradoxically there are potential unintended consequences associated with this. For example, a greater number or variety of destinations can draw people into an area which can increase crime levels [31],[42] and perceptions of crime risk [48],[49]. This largely stems from the opportunistic nature of many offences, where crimes are often committed as opportunities arise, whilst individuals travel to and carry out their routine activities [34]. Just as walkable neighbourhood characteristics often occur concurrently (i.e., residential density, street connectivity and mixed land-uses) [50]; crime may be a ‘part of modern life’ that is intertwined with certain land-uses and physical characteristics of places [34],[42]. Indeed, higher levels of crime might be a necessary trade-off to live in a more walkable, potentially vibrant neighbourhood.

In this study, there was greater attenuation of the association between personal crime and walking when destinations were limited to those likely to serve alcohol. This might be expected, given the strong links between alcohol outlets and crime, particularly violent offences committed in the public realm [37]–[39]. Nonetheless, it was apparent that neither alcohol-related destinations, nor the offences related to them, had any negative impact on walkers, perhaps suggesting a more nuanced relationship between the social and built realm of local communities. For example, while alcohol outlets can lead to public intoxication, violence, street disturbances, and a range of other social problems [38],[51], a well-run venue can also provide an important meeting place for social interaction and can potentially be an asset to a local community [52]. This role as a ‘third place’ [53] may offset or balance some of the negative consequences of such venues.

This study was set in a relatively safe city, largely dominated by low-density suburban development, often with poor access to shops, services and public transport [54]. Thus, it is possible that the crime levels were insufficient to negatively impact walking. One might hypothesise that different findings would be evident in areas with higher crime levels, where residents are genuinely more vulnerable; however no clear pattern emerges from the studies set in lower socio-economic or deprived communities [18],[20],[27],[46]. The association between objective crime and physical activity is likely to be more complex, with numerous other factors impacting the association, including the age of participants [21], individual and area-level socio-economic status [18],[20],[46], perceptions of neighbourhood trust and cohesiveness [27], and as demonstrated in our study, the characteristics of a walkable environment.

This study focused exclusively on the association between objective measures of crime and walking, as subjective perceptions of crime were not available. However, previous research in the same relatively safe city highlights the different findings that can stem from subjective crime measures, particularly emotional responses to crime [13],[26],[55]. While the current study found that objective crime had little bearing on whether residents’ walk, our recent longitudinal study found that fear of crime (i.e., a subjective, emotional response to crime) had a significant, and sizeable, negative influence on walking. For every increase in fear of crime on a five-point scale, total walking reduced by approximately 22 minutes per week [13]. The contradiction between these findings accentuates the disconnect between objective and subjective measures of crime. There are additional complexities associated with subjective crime measures, and the actual incidence of crime may be just one among many factors that impact perceptions of crime or emotional responses to crime [9],[56],[57]. Indeed, it has been suggested that fear of crime may capture other, more nebulous anxieties about life, which are ‘projected onto a knowable and name-able fear’ [58], p.261. In relatively low crime settings, emotional responses to crime may ultimately prove a more powerful influence on walking than actual crime, but notably, alleviating fear of crime may be better achieved by targeting the individual, social and physical environmental factors that can impact fear, such as social connections and neighbourhood upkeep, rather than crime reduction *per se*.

This study has a number of limitations. First, we focused on the associations between objective (or reported) crime and walking, and crime categories were limited to burglary and personal crime in public space. These data may underrepresent serious offences due to embarrassment or concerns about retaliation, and there may be a discrepancy in the reporting of crime to police by socio-economic status (i.e., crime is more likely to be reported in high income areas) [24]. Moreover, the offences examined in this study are not necessarily ‘visible’, and it is plausible that crime might only deter walkers in neighbourhoods where there are also visual indicators of crime (e.g., graffiti, vandalism, litter, drug paraphernalia). Second, we examined self-report walking frequency but are unable to identify whether walking was undertaken locally. It is possible that residents in higher crime neighbourhoods walk in other, safer environments. Third, this is a cross-sectional study, and there-fore participants who prefer to walk may choose to live in more walkable neighbourhoods, perhaps with a tacit awareness that there may also be more crime [48]. Despite these limitations, the study also had several strengths, including a large population representative sample, and individual-level environment measures for each participant’s 400 m and 1600 m neighbourhoods.

## Conclusion

Studies investigating objective measures of crime as a barrier to walking have produced mixed results. One possible explanation is that the built environment attributes that support walking, particularly walking for transport, have also been linked with more crime. In this study we identified a positive association between objective crime and walking, which attenuated after accounting for the presence of local destinations, and more specifically, destinations that serve alcohol. Our findings suggest that the local destinations that are inherent to a walkable neighbourhood have the potential to both encourage walkers and attract crime, and that this may account for some of the non-significant or counter-intuitive findings observed in the literature. Ultimately, crime may be another attribute of the neighbourhood environment that is intertwined with walkable neighbourhood design. An acceptance of higher levels of crime might be a necessary concession for those residents living in a more walkable, potentially vibrant neighbourhood.

## Competing interests

The authors declare that they have no competing interests.

## Authors’ contributions

SF conducted the analyses, interpreted the data and drafted the manuscript. MK and BGC advised on data analysis and interpretation of results. KV prepared the data. All authors contributed to the study conception and design, provided input into to manuscript drafts and approved the final manuscript.
